# Protection effect of gut microbiota composition and acetate absorption against hypertension-induced damages on the longevity population in Guangxi, China

**DOI:** 10.3389/fnut.2022.1070223

**Published:** 2023-01-16

**Authors:** Qinren Zhang, Ning Meng, Yu Liu, Haiyan Zhao, Zhengtao Zhao, Dan Hao, Ruiding Li, Kunchen Han, He Li, Jinke Ma, Xiaohan Yu, Zhongquan Qi, Quanyang Li

**Affiliations:** ^1^College of Light Industry and Food Engineering, Guangxi University, Nanning, China; ^2^Medical College, Guangxi University, Nanning, China; ^3^Department of Pharmacology and Nutritional Sciences, University of Kentucky, Lexington, KY, United States

**Keywords:** gut microbiota, hypertension, longevity, short-chain fatty acids, urine metabolites

## Abstract

**Introduction:**

Recent evidence supports a role for the gut microbe-metabolites in longevity. However, the phenomenon of hypertension is more common in the longevity area and whether hypertension is associated with longevity remains unclear. Here, we hypothesize that the levels of gut microbiota, SCFAs, and urine metabolites were different between hypertension elderly and hypertension longevity.

**Methods:**

We recruited 46 elderly volunteers from Donglan County, Guangxi, and 32 were selected and included in the experiment. The subjects with hypertension were divided into two groups according to age, Hypertension Elderly (HTE, aged 70.5 ± 8.59, *n* = 19) and Hypertension Longevity (HTL, aged 100 ± 5.72, *n* = 13). The gut microbiota, SCFAs, and urine metabolites were determined by three-generation 16S rRNA full-length sequencing, GC-MS, and ^1^H-NMR, respectively.

**Results:**

Compared with the HTL group, the HTE group had higher levels of hypertension-related genera *Klebsiella* and *Streptococcus*, while having lower levels of the SCFA-producing genera *Bacteroides*, *Faecalibacterium*, and *Alistipes*. Based on LEFse analysis, *Klebsiella pneumoniae*, *Lactobacillus gasseri*, *Streptococcus salivarius, Ruminococcus, Actinomyces, Rikenellaceae, f_Saccharimonadaceae, Clostridium perfringens*, and *Bacteroids, Faecalibacterium prausnitzii, Parabacteroides, Alistipes* were biomarkers that showed significant differences between the groups. In addition, the microbial pathways associated with *K. pneumoniae* and *E. coli* may promote hypertension, while *A. muciniphila* may play a role in reversing the development of hypertension in long-lived elderly. Metabolomics revealed that HTL contained a lower concentration of fecal acetate and propionate than HTE, while it contained a higher concentration of serum acetate and urine acetate. Furthermore, their immune cells exhibited no significant changes in SCFAs receptors.

**Conclusion:**

Although long-lived elderly have extremely high systolic blood pressure, their unique gut microbiota composition and efficient acetate absorption in the colon may offset the damages caused by hypertension and maintain healthy homeostasis.

## Graphical Abstract

Summary of the study findings. 
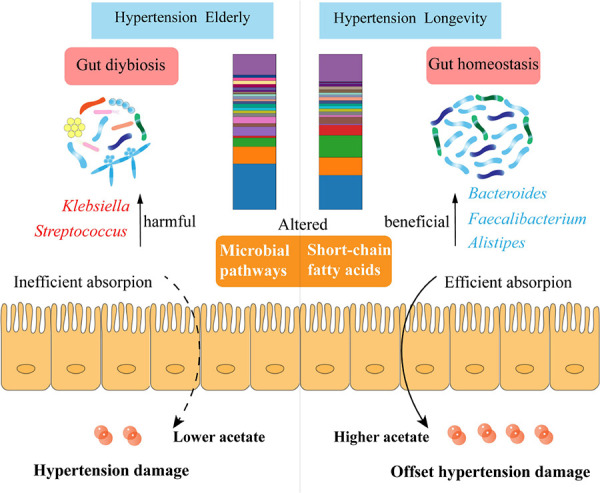

## Introduction

Hypertension is a major cause of cardiovascular disease and has become a public health problem worldwide. The pathogenesis of hypertension involves multiple factors, including genetics, environment, hormones, and inflammation (1). There is increasing evidence that the gut microbiota plays an essential role in the development and pathogenesis of hypertension (2). The gastrointestinal tract is the largest immune interface with the environment in the human body. An association between hypertension and gut microbiota has been found in human and animal models (3). In hypertensive patients, the α-diversity of gut microbiota was decreased and highly correlated with gram-negative flora, including increased genera *Klebsiella*, *Streptococcus*, *Parabacteria*, *Desulfovibrio*, and *Prevotella* (4), while some short-chain fatty acid-producing bacteria, such as *Faecalibacterium*, *Roseburia spp* will be reduced (5). It was also found that high blood pressure is correlated to gut dysbiosis in spontaneously hypertensive rats (6). Germ-free mice transplanted with fecal bacteria from hypertensive humans developed gut microbiota similar to their donors and increased systolic and diastolic blood pressure after 8 weeks (2). Together, these studies suggest that hypertensive patients have disturbed gut microbiota and an increase in some of the characteristic genera associated with hypertension.

Changes in gut microbiota can lead to changes in microbial-derived metabolites. Among them, short-chain fatty acids (SCFAs), as the product of bacterial fermentation of dietary fiber, are closely related to health (7). SCFAs are produced and absorbed in the colon and excreted in the feces, so fecal SCFAs reflect levels that cannot be absorbed in the colon (8). Previous research indicated that the efficient absorption of acetate, propionate, and butyrate in the colon decreased the blood pressure in hypertensive experimental animals (9, 10), which was further confirmed by the higher levels of fecal SCFAs in hypertensive patients (11). Therefore, fecal SCFAs can be used as a surrogate index of the absorption of SCFAs in the body.

Numerous studies have used the longevity population to study the relationship between gut microbiota and longevity (12–14). The gut microbiota of centenarians is rich in health-related bacteria such as *Akkermansia*, *Christensenellaceae*, and *Bifidobacterium* (15), which produce high levels of specific secondary bile acids and effectively inhibit gram-positive pathogenic bacteria (16). It has a higher ability of glycolysis and fermentation to generate short-chain fatty acids (17). However, long-lived elderly and their offspring have widespread hypertension (18). Most of them live in remote mountain villages, such as Bama in Guangxi (19), Sardinia (17), rural Korea (20), and Moscow (21), etc., are rarely affected by the modern urbanization environment. Bama, Donglan, and Fengshan are the three most famous longevity populations in Guangxi, and which prevalence of the three counties was 35.6, 31.4, and 28.5 centenarians/100,000 citizens in 2019, respectively (13). In our previous study, 66.7% of the population aged >60 in this longevity area had SBP≥140 mmHg (22). This co-existence of longevity and hypertension is well worth investigating. We hypothesize that these long-lived populations have unique gut microbiota and microbial-derived metabolites that offsets hypertension damage and thus maintain healthy homeostasis.

Based on the above, this study conducts a cross-sectional study of gut microbiota, urine metabolites, SCFAs, and their receptors in longevity areas in Donglan County, Guangxi, through three generations of 16S full-length sequencing, metabolomics, and qPCR, respectively. Analyzing the particularity of gut microbiota composition of long-lived people with hypertension, and combing the corresponding metabolites to explain the relationship between longevity and hypertension, can provide a reference for the prevention and treatment of hypertension.

## Materials and methods

### Participant recruitment and study groups

This research was approved by the Ethics Committee of Guangxi University (approval number: gxdxyxll05). For the experiment design, 46 subjects from Donglan County, Hechi City, Guangxi, were recruited, and all subjects signed written informed consent. The general conditions of the research subjects were recorded, including their age, gender, height, weight, and blood pressure of the subjects. The age information was verified by comparing the ID card or household registration. Clinical and medical history were collected based on the self-report and physical examination reports (Supplementary Table 1). Blood pressure (BP) was measured using a calibrated arm electronic sphygmomanometer (Omron). When measuring blood pressure, subjects were asked to sit still for 5 min with their feet flat on the floor without crossing them, and their upper arms were relaxed and placed on a table at the same level as the heart. Two measurements were taken 1 min apart, and if systolic BP was >15 mm Hg apart or diastolic BP > 10 mm Hg apart, a third measurement was performed and averaged. Inclusion criteria: (i) age ≥ 60 years, and either sex, (ii) body mass index (BMI) 17–25 kg/m^2^, (iii) systolic blood pressure (SBP) ≥ 140 mmHg, and not using BP-lowering medication, (iv) from autochthonous families, none was coming from other regions originally. Exclusion criteria: (i) history of chronic medical conditions (diabetes, gastroenteritis, chronic kidney disease, morbid obesity, chronic pancreatitis, or other malabsorption disorder), (ii) use of probiotics, prebiotics, or antimicrobial medication (antibiotic or antifungal treatments) 1 year before sampling. Four participants were excluded due to high BMI, four were excluded due to the SBP lower than 140 mmHg, and five were excluded due to chronic medical conditions. One participant dropped out due to unwillingness to continue (Supplementary Figure 1). Following criteria, a total of 32 participants remained: 19 participants aged 60–90 were divided into Hypertension-Elderly (HTE, average 70.5) group, and 13 participants aged 92+ were divided into Hypertension-Longevity (HTL, average 100). The characteristics of the participants are shown in Table 1.

**TABLE 1:** Demographics and clinical characteristics of participants.

	Hypertension elderly	Hypertension longevity	Total	*p*-value
	(*N* = 19)	(*N* = 13)	(*N* = 32)	
**Sex**				
Male	10 (52.6%)	4 (30.8%)	14 (43.8%)	
Female	9 (47.4%)	9 (69.2%)	18 (56.3%)	
Age (years)				<0.001
Mean (SD)	70.5 (8.59)	100 (5.72)	82.6 (12.6)	
Median [min, max]	70.0 [60.0, 88.0]	101 [92.0, 109]	81.5 [60.0, 109]	
BMI				0.323
Mean (SD)	21.7 (2.32)	20.9 (2.26)	21.4 (2.29)	
Median [min, max]	22.0[17.3, 24.8]	21.5 [17.1, 24.2]	21.6 [17.1, 24.8]	
SBP (mmHg)				0.305
Mean (SD)	154 (14.8)	163 (22.6)	157 (18.5)	
Median [min, max]	148 [140, 190]	163 [141, 207]	148 [140, 207]	
DBP (mmHg)				0.223
Mean (SD)	90.1 (9.26)	86.9 (6.70)	88.8 (8.35)	
Median [min, max]	90.0 [69.0, 108]	88.0 [75.0, 98.5]	89.0 [69.0, 108]	
Pulse				0.472
Mean (SD)	75.2 (9.07)	73.7 (8.47)	74.6 (8.72)	
Median [min, max]	74.0 [59.0, 91.0]	71.0 [61.0, 95.5]	73.3 [59.0, 95.5]	

Data are shown as mean (SD) or numbers and percentages. *P*-value from Mann–Whitney. *P* < 0.05 is statistically significant. BMI, body mass index; SBP, systolic blood pressure; DBP, diastolic blood pressure.

### Dietary assessment

Participants’ dietary and nutritional status was assessed using a semi-quantitative food frequency questionnaire (FFQ), which was recorded for consecutive 7-days at home (23, 24). All participants were asked to adopt the mode of individual dining and were urged to maintain their usual eating patterns at home during the dietary survey. Trained investigators used electronic food scales, measuring cups, and spoons to weigh and record each food and drink the participants consumed. For unmeasurable food, the standard portion size was used. Qualified dietitians evaluated and verified the dietary data at a dietary survey location for quality control purposes. Dietary data was converted to nutritional consumption levels according to the 2022 Dietary Guidelines for Chinese Residents. The average daily nutritional intake was determined by multiplying the food consumed (in grams) or the portion size consumed by the nutrient content per 100 g of the food listed in the guideline (25).

### 16S rRNA third-generation full-length sequencing of gut microbiota

Our study followed guidelines for gut microbiota studies in hypertension (26) and the STORMS (Strengthening the Organization and Reporting of Microbiome Studies) reporting (27) (Supplementary Table 2). Fecal samples of the participants were collected by themselves or their family members with an icebox at home in sterilized empty tubes (for SCFA determination) or tubes containing RNAlater (Thermo Scientific) for microbial DNA extraction. Morning urine samples were collected in sterilized empty tubes with an icebox. Tubes were immediately stored in a small car refrigerator (Alpicool, A30) at −20°C for <24 h. Then, the samples were transported to the laboratory by professionals and stored at −80°C after dispensing into 2 mL sterilized EP tubes until analysis. DNA was extracted using the Stool Genomic DNA Extraction Kit (Solarbio) according to the manufacturer’s instructions. After extraction, PCR amplification of the full-length region of bacterial 16S rRNA was performed using universal primers 27F (5′-AGRGTTTGATYNTGGCTCAG-3′), 1492R (5′-TASGGHTACCTTGTTASGACTT-3′), and purified with magicpure size selection DNA beads (TANGEN Biotech Corporation Ltd., Beijing, China).

Subsequently, the quality inspection was performed on the formed sequencing library, and processing, including barcode recognition, was performed on the high-quality circular consensus sequencing (CCS) sequence obtained. The generated optimization CCS was clustered at 97% similarity (USEARCH, version 10.0), and its species classification was obtained based on the operational taxonomic unit (OTU) sequence composition. The platforms of 16S: Silva database and RDP Classifier were used to analyze species annotation and taxonomy and gut microbiota diversity. Alpha diversity analysis was performed to examine the species richness and diversity within a single sample, and each sample’s ACE, Chao, Shannon, and Simpson indices were calculated. The differences in the community composition and structure of different samples were compared through Beta diversity analysis. Finally, Metastats analysis was carried out to compare the significant differences between the groups at the genus level, and linear discriminant analysis effect size (LEfSe) analysis was used to screen biomarkers that were statistically different between the groups (criteria: LDA score > 3.5). The raw sequencing data generated in this study have been deposited to the NCBI SRA database (BioProject: PRJNA888351).

### Fecal short-chain fatty acid measurement

Serum SCFAs were measured in 400 μL, and fecal SCFAs were measured from 0.2 g of fecal sample, all in triplicates. For the fecal sample extraction, 0.2 g of −80°C frozen feces were mixed with 2 mL ultra-pure water in a centrifuge tube. Samples were shaken for 2 min and centrifuged at 12,000 rpm for 20 min (4°C). After centrifugation, 1 mL supernatant was mixed with 0.25 mL of 25% metaphosphoric acid, kept in ice bath at −20°C for 2 h. After thawed, the samples were centrifuged at 12,000 rpm for 10 min (4°C). The supernatant (1 mL) was mixed with 1 mL ethyl acetate, vortexed and centrifuged for 12,000 rpm for 5 min (4°C) to obtain the clear organic layer, which was aspirated and filtered through an organic green membrane into the cold GC glass vials for analysis. For the serum sample extraction, 400 μL serum was mixed with 50 μL of 25% metaphosphoric acid and kept in ice bath at −20°C for 2 h. Follow steps were similar to fecal extraction and took care to add proportion. The analysis of acetate, propionate, isobutyrate, butyrate, isovalerate, and valerate was performed by gas chromatography-mass spectrometry (Agilent 7890A Series) using a capillary DB-Wax column (30 m, 0.25 μm, 0.25 μm; SGE, Cromlab SL, Barcelona, Spain), coupled with a flame ionization detector (GC-FID). The column temperature was programmed at 80°C, reached 110°C at a rate of 10°C/min, maintained for 1 min, then increased to 160°C at 4°C/min, and maintained 1 min (total run time 17.5 min). Helium was the carrier gas (0.5 mL/min). The injection was carried out with a split injector (1:100) at 220°C, the detector temperature was 250°C, and 1 μL of the solution was injected into the GC-FID system. The SCFAs was identified according to standard compounds’ retention time of standard compounds (acetate, propionate, isobutyrate, butyrate, isovalerate, and valerate; Sigma-Aldrich).

### Analysis of urine metabolites based on ^1^H-nuclear magnetic resonance

Aliquots of 500 μL of urine were transferred into a 2.0 mL centrifuge tube and added with 500 μL of 100 mM phosphate buffer containing 0.05% TSP-labeled heavy water (10% heavy water, K_2_HPO_4_/NaH_2_PO_4_, PH = 7.4). The mixtures were vortexed and centrifuged (15 min, 12,000 × rpm, 4°C) to obtain the urine supernatant.

#### ^1^H-NMR analysis

The prepared urine supernatant (550 uL) was placed on the Bruker AVANCE 500 MHZ nuclear magnetic resonance spectrometer of the Prodigy liquid nitrogen cryogenic probe for NMR spectrum detection. The pre-saturation method suppressed the water peak, and the NOESY pulse sequence was used. The specific setting parameters are sampling times NS = 64, temperature 25°C, spectrum width SWH = 10,000 Hz, measurement frequency SF = 500.13 MHz, relaxation delay D1 = 2 s, number of sampling points TD = 65,536, sampling time AQ = 3.277 s, mixing time 0.1 s, O1P = 4.7ppm, FID resolution 0.245.

#### ^1^H-NMR spectrum processing and data analysis

The Fourier transform of the fecal ^1^H-NMR spectrum was performed using MestReNova NMR data processing software (Mestrelab Research, Spain), and the phases and baseline were adjusted. The chemical shift of TSP (δ 0.0) was taken as the standard to calibrate the chemical shift. The water peak within the range of δ 4.70–5.00 was excised, and the NMR spectra with δ 0.60–9.00 were segmented with equal width in the unit of 0.01 for segment integration, and the data were normalized. The above data were stored in Excel tables for statistical analysis.

### Blood expression of SCFA receptors and transporters

The three main SCFA-sensing receptors GPR41 (FFAR3), GPR43 (FFAR2), and GPR109A (HCAR2) have a role in cardiovascular dysfunction (28). As these receptors are highly expressed in immune cells (29), we quantified the mRNA expression of the 3 receptors in circulating immune cells in 30 participants. Since no blood samples were collected from the participants, we selected 30 samples(15 in HTE and 15 in HTL) from long-lived populations with hypertension characteristics collected earlier (23). The basic information of these samples is presented in Supplementary Table 3. Whole blood was treated with Red Blood Cell Lysis Buffer (ThermoFisher Scientific), and RNA was extracted using the Total RNA Extraction Kit (Solarbio). RNA was quantified in a Nanodrop Spectrophotometer, and the first-strand complementary synthesis reaction (cDNA) was made using the BeyoRT™ III First Strand cDNA Synthesis Kit (Beyotime). SYBR Green I was used in a Roche LIGHTCYCLER 96 Real-time quantitative PCR (qPCR) system (Roche Diagnostics Co., Ltd., Basel, Switzerland), with GAPDH as housekeeping genes (Supplementary Table 4). All expression experiments were run in duplicates, and significance was assessed by the 2–ΔΔCT method.

### Statistics and analysis

Microbiome data were analyzed as explained above. Graphs were generated using GraphPad Prism version 9.0. Adobe Illustrator version 2020 was used to figure combinations and graphical abstract drawings. All the analyses were performed using SPSS version 26.0 (SPSS Inc., United States) and a 5% significance level. The data were presented as mean ± SD values. The Mann–Whitney U test was used to determine the statistical difference between the two groups gut microbiota, SCFAs, and urine metabolites. A spearman correlation test was conducted to explore correlations between the differential genus and significant microbial pathways and nutrient intake. Pearson correlation analysis was performed to examine correlations between blood pressure and levels of SCFAs, and urine metabolites, after adjustment for age and BMI. Further analyses were conducted using simple linear regression models for BP, acetate, butyrate, and propionate.

## Results

### Nutrient intake in subjects

The nutrient intake levels in the two groups are presented in Table 2. The two groups had significantly different nutrient intake levels. Notably, the level of energy, fat, saturated fatty acid (SFA), monounsaturated fatty acids (MUFA), polyunsaturated fatty acids (PUFA), carbohydrate, thiamine, riboflavin, vitamin E, folic acid, phosphorus, potassium, calcium, zinc, and manganese were significantly lower in subjects in the HTL compared with the group HTE (*p* < 0.05). Furthermore, the intake of protein, dietary fiber, cholesterol, vitamin C, vitamin K, nicotinic acid, phosphorus, sodium, magnesium, iron, and selenium were at the same level between the groups. In addition, there were no significant differences in the number of food items consumed by participants, probably due to the similar dietary habits of the participants in the longevity areas. The spearman correlation was used to calculate the relationship between genus and nutrient intake (Supplementary Figure 2). The genus of *Faecalibacterium* was positively correlated with sodium, whereas the genus of *Subdoligranulum* was positively correlated with energy, fat, SFA, PUFA, vitamin A, thiamine, riboflavin, vitamin E, phosphorus, magnesium, calcium, iron, and zinc. *Clostridium* was negatively correlated with vitamin A, iron, and zinc. *Christensenellaceae_R7_group* had a negative correlation with vitamin C.

**TABLE 2:** The daily average intake of nutrients in the HTE and HTL.

	HTE	HTL	*p*
**Nutrients**			
Energy(Kcal)	1514.69 ± 215.64	1315.94 ± 218.92	0.011
Protein(g)	61.34 ± 12.22	54.31 ± 11.94	0.099
Fat(g)	36.09 ± 11.25	25.97 ± 10.76	0.008
SFA (g)	9.02 ± 2.80	6.54 ± 2.83	0.013
MUFA (g)	8.24 ± 3.41	5.21 ± 3.43	0.014
PUFA (g)	12.47 ± 4.03	8.84 ± 4.56	0.009
Carbohydrate(g)	114.47 ± 21.08	95.74 ± 17.63	0.016
Dietary Fiber(g)	29.84 ± 5.44	27.09 ± 2.44	0.343
Cholesterol (mg)	105.49 ± 43.22	89.50 ± 39.29	0.170
Vitamin A (μgRE)	847.94 ± 151.54	751.17 ± 97.72	0.071
Thiamine (mg)	0.69 ± 0.11	0.58 ± 0.10	0.010
Riboflavin (mg)	1.07 ± 0.18	0.92 ± 0.17	0.010
Vitamin C (mg)	51.14 ± 12.71	41.51 ± 9.12	0.147
Vitamin E (mg)	9.38 ± 3.73	6.23 ± 3.57	0.020
Vitamin K (μg)	84.72 ± 19.90	82.75 ± 16.81	0.677
Folic acid (μg)	237.43 ± 54.80	190.97 ± 40.69	0.018
Nicotinic acid (mg)	15.85 ± 4.15	14.51 ± 3.58	0.170
Phosphorus (mg)	650.13 ± 127.38	550.49 ± 124.28	0.049
Sodium (mg)	466.55 ± 209.35	360.47 ± 199.06	0.158
Potassium (mg)	2042.27 ± 330.72	1809.42 ± 262.95	0.041
Magnesium (mg)	210.18 ± 36.94	185.43 ± 29.66	0.223
Calcium (mg)	191.36 ± 34.67	159.92 ± 33.93	0.022
Iron (mg)	21.03 ± 33.85	21.45 ± 33.92	0.108
Zinc (mg)	6.35 ± 2.04	5.31 ± 2.16	0.037
Selenium (μg)	16.04 ± 6.99	11.74 ± 7.47	0.099
Manganese (mg)	3.77 ± 1.08	2.91 ± 1.12	0.008
**Diversity of food**			
Number of different food items consumed by participants, *item/d	12.47 ± 0.77	11.69 ± 1.44	0.170

*Only accounting for food items with >10 g that participants consumed both within and outside of the study. Condiments were not included. SFA, saturated fatty acid; MUFA, monounsaturated fatty acids; PUFA, polyunsaturated fatty acids.

### Analysis of gut microbiota characteristics between group

Through high-throughput sequencing and sequence screening, 400,724 CCS sequences were obtained through the Barcode identification of 32 samples after sequencing. Each sample generated at least 10,254 CCS sequences, with an average of 12,523 CCS sequences. Operational taxonomic units (OTUs) were divided according to 97% sequence similarity, and a total of 360 OTUs were obtained for subsequent analysis. Through the Shannon curve and the rank abund curve, it was found that the number of OTUs in all samples increased with the increase in sequencing amount and eventually plateaued, indicating that the sequencing volume is sufficient to achieve the ideal sequencing depth (Supplementary Figure 3). The shared and unique OTUs within and between groups were analyzed and plotted as Venn diagrams. HTL had six shared OTU features, HTE had three shared OTU features, and 306 shared OTU features between the two groups (Supplementary Figure 4). Alpha diversity was assessed by five parameters and showed no significant difference between HTE and HTL (Supplementary Table 5).

**FIGURE 1:**
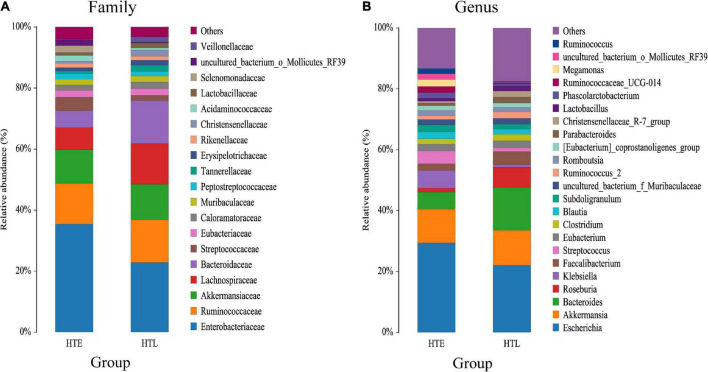
Bar chart of the relative abundance (%) gut microbiota profiles. The taxa with average relative abundance above 1% are displayed at the family **(A)**, and genus **(B)** levels.

A total of 11 phylum were dominated by *Firmicutes*, *Proteobacteria*, *Bacteroidetes*, and *Verrucomicrobiota*, which accounted for 97.32% (HTE) and 99.01% (HTL), respectively. The ratio of *Firmicutes* to *Bacteroidetes*(F/B) of HTL (2.14) was significantly lower than HTE (3.71). Figure 1 shows species with relative abundances greater than 1% at the family and genus levels. Both groups were dominated by five families, *Enterobacteriaceae*, *Ruminococcaceae*, *Akkermansiaceae*, *Bacteroidaceae*, and *Lachnospiraceae*, with cumulative abundances of 72.61% (HTE) and 75.90% (HTL), respectively. Compared with HTL, the HTE group had much higher levels of the hypertension-related bacteria genera *Klebsiella* and *Streptococcus* (+655% and +242%), while it had lower levels of SCFA-producing bacteria *Bacteroides* (–60%), *Roseburia* (–80%), *Faecalibacterium* (–50%), and *Alistipes* (–79%), respectively. Table 3 further presents the significant difference between the groups in taxa at the genus and species level. The above results showed that the HTE experienced gut dysbiosis, while HTL were abundant in SCFA-producing bacteria. These results are consistent with the commonly reported hypertensive gut genera in addition to identifying two new health-associated bacterial genera (Supplementary Table 6).

**TABLE 3:** The significant difference genus and species between HTE and HTL.

Taxa	Median, range^1^ (%)		*p*-value
	HTE	HTL	
**Genus**			
*Bacteroides*	5.57, 0.57–10.57	8.06, 4.61–22.73	0.033
*Klebsiella*	3.73, 0.55–8.53	0.31, 0.03–1.57	0.005
*Streptococcus*	0.67, 0.23–5.45	0.28, 0.02–0.66	0.059
*Faecalibacterim*	1.02, 0.03–2.55	2.91, 0.20–7.49	0.03
*Parabacteroides*	0.18, 0.02–0.42	0.40, 0.16–3.04	0.037
*Ruminococcus*	0.64, 0.18–2.63	0.13, 0.06–0.45	0.018
*Alistipes*	0.11, 0.04–0.38	0.88, 0.39–2.078	<0.001
*Erysipelotrichaceae_UCG-003*	0.02, 0.00–0.34	0.33, 0.03–1.22	0.041
**Species**			
*Klebsiella pneumoniae*	3.73, 0.55–8.53	0.31, 0.03–1.57	0.005
*Faecalibacterium prausnitzii*	1.02, 0.03–2.55	2.91, 0.20–7.49	0.03
*Streptococcus salivarius*	0.24, 0.11–1.05	0.01, 0.00–0.33	0.007
*Clostridium perfringens*	0.05, 0.01–0.15	0.00, 0.00–0.04	0.049
*g_Erysipelotrichaceae_UCG-003*	0.02, 0.00–0.34	0.33, 0.03–1.22	0.041
*Bacteroides massiliensis*	0.00, 0.00–0.09	0.37, 0.02–0.73	0.02
*Bacteroides caccae*	0.01, 0.00–0.03	0.42, 0.06–0.79	0.008
*Alistipes putredinis*	0.03, 0.00–0.22	0.31, 0.04–1.16	0.022
*Alistipes finegoldii*	0.00, 0.00–0.04	0.20, 0.09–0.83	<0.001
*Parabacteroides_sp*	0.04, 0.00–0.16	0.22, 0.07–0.70	0.018
*Lactobacillus mucosae*	0.01, 0.00–0.04	0.00, 0.00–0.00	0.049

^1^Range represents the up quartile and the lower quartile.

**FIGURE 2:**
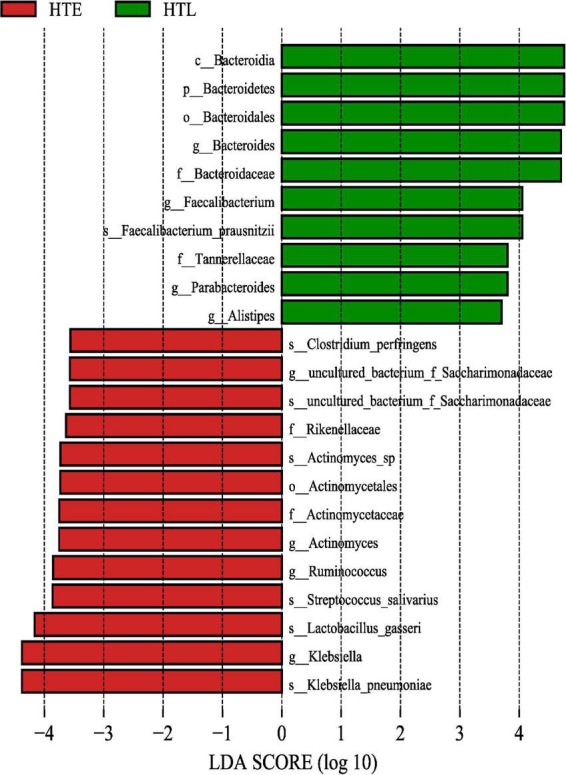
The gut microbiome taxa linear discriminant analysis effect sizes. Predicted gut microbiome taxa that are different between hypertension elderly and hypertension longevity, with a linear discriminant analysis (LDA) score of least 3.5.

The LEfSe method was used to analyze and screen out the biomarker with an LDA score > 3.5. A total of 23 differential species were found between the two groups, *Klebsiella pneumoniae*, *Lactobacillus gasseri*, *Streptococcus salivarius*, *Ruminococcus*, *Actinomyces*, *Rikenellaceae*, *f_Saccharimonadaceae*, *Clostridium perfringens* in the HTE and *Bacteroids, Faecalibacterium prausnitzii*, *Parabacteroides*, *Alistipes* in the HTL group, respectively (Figure 2).

### Hypertension-related microbial pathways

**FIGURE 3:**
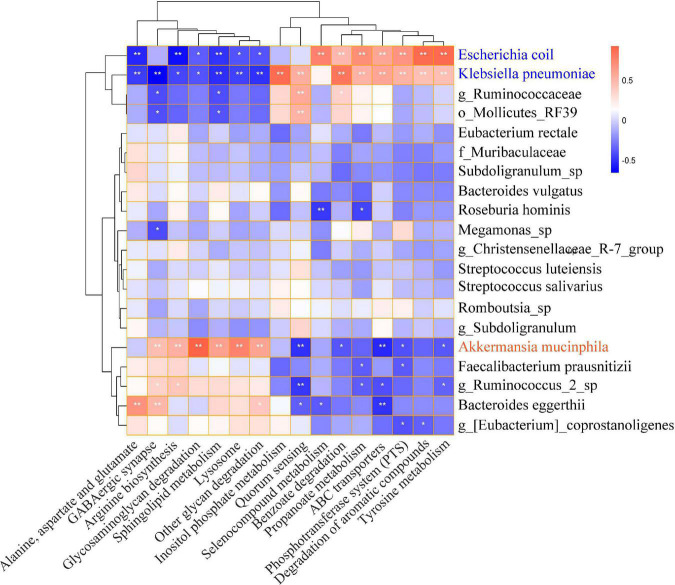
Heat map of Spearman correlation between significantly microbial pathways and top 20 abundance species. The *X*-axis indicates the significantly different microbial pathways, and the *Y*-axis indicates the top 20 levels of species. Red represents positive correlation, and blue represents negative correlation. The stronger the color, the closer the number is to the Spearman correlation value of 1 or –1, *indicates significant correlation, *p < 0.05, **p < 0.01.

PICRUSTs2 was performed to predict the functional prediction of KEGG microbial pathways in gut microbiota, and a total of 248 Class3 microbial pathways were obtained. The biosynthesis of secondary metabolites, biosynthesis of antibiotics, microbial metabolism in diverse environments, and biosynthesis of amino acids were significantly enriched in both HTE and HTL groups. There were 114 Class3 microbial pathways with relative abundance greater than 0.1% and 16 microbial pathways were significantly different between HTE and HTL groups, including ABC transporters, alanine, aspartate and glutamate metabolism, arginine biosynthesis, benzoate degradation, degradation of aromatic compounds, GABAergic synapse, glycosaminoglycan metabolism, inositol phosphate metabolism, lysosome, other glycan degradation, phosphotransferase system (PTS), propanoate degradation, quorum sensing, selenocompound metabolism, sphingolipid metabolism, and tyrosine metabolism (Supplementary Table 7). The correlations between the top 20 species and 16 significant microbial pathways were further analyzed by Spearman correlation (Figure 3). Interestingly, the association of the hypertensive marker species *Klebsiella pneumoniae* with microbial pathways was consistent with *Escherichia coli*, but the correlation was reversed for *Akkermansia muciniphila*.

### Fecal short-chain fatty acid levels

**FIGURE 4:**
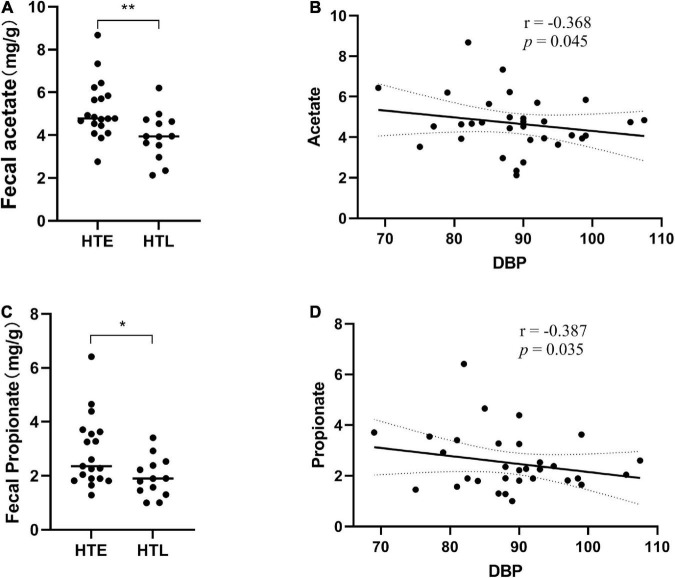
Fecal levels of gut microbial metabolites short-chain fatty acids (SCFAs) in association with hypertension. **(A)** Acetate, **(B)** the correlation analysis between acetate and diastolic blood pressure (DBP), **(C)** propionate, **(D)** the correlation analysis between propionate and diastolic blood pressure (DBP). Sample size: *n* = 19 hypertension elderly and 13 hypertension longevity. *Indicates *p* < 0.05, **p < 0.01.

Six short-chain fatty acids (SCFAs) in fecal samples, namely acetate, propionate, isobutyrate, butyrate, isovalerate, and valerate, were qualitatively detected by GC-MS. Acetate (*p* = 0.008) and propionate (*p* = 0.033) were significantly lower in HTL feces than in HTE (Figures 4A, C). Correlation analysis showed that there was no correlation between SBP and SCFAs, but DBP was negatively correlated with acetate (*r* = −0.368, *p* = 0.045) and propionate (*r* = −0.387, *p* = 0.035) (Figures 4B, D). There were significant correlations between SCFAs, and acetate was significantly positively correlated with propionate (*r* = 0.708, *p* = 0.000), butyrate (*r* = 0.545, *p* = 0.002), and valerate (*r* = 0.453, *p* = 0.012) (correlation results after adjusted for age and BMI).

### Statistical analysis of urine metabolites

**FIGURE 5:**
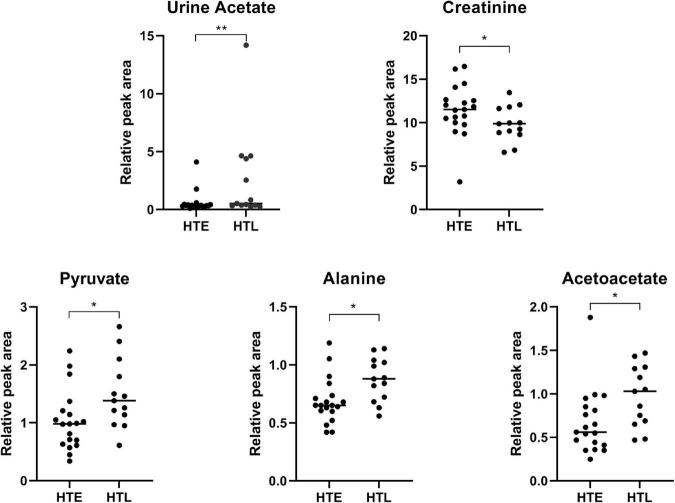
Urine metabolites with significant differences between groups. Sample size: *n* = 19 hypertension elderly and 13 hypertension longevity. *Indicates *p* < 0.05. **P < 0.01.

Through urine ^1^H-NMR metabolomic analysis, 36 metabolites were identified (Supplementary Figure 5). Compared with HTE, urine acetate (*p* < 0.001), pyruvate (*p* = 0.022), alanine (*p* = 0.014), and acetoacetate (*p* = 0.010) were significantly higher in HTL, while creatinine was significantly lower (*p* = 0.041) (Figure 5). Supplementary Table 8 shows the urine metabolites significantly associated with SBP and DBP. In particular, N-acetyl glycoprotein was negatively correlated with both SBP and DBP (correlation results were adjusted by age and BMI).

### SCFAs and receptors

**FIGURE 6:**
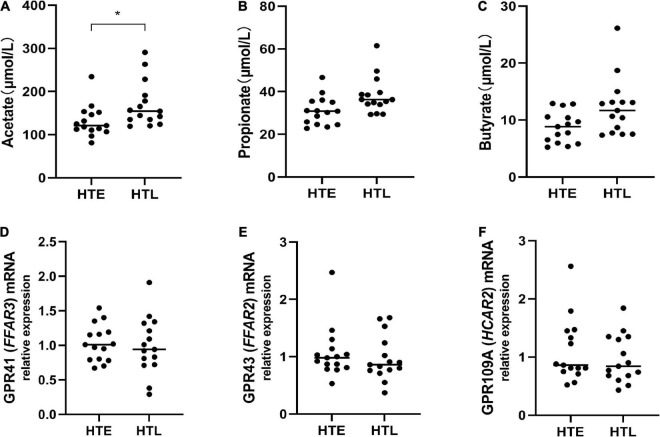
Plasma levels of short-chain fatty acids (SCFAs) and relative expression of short-chain fatty acids (SCFAs)-sensing receptors. **(A)** Acetate, **(B)** propionate, **(C)** butyrate, the mRNA expression in white blood cells between groups, while there are no change in GPR41 **(D)**, GPR43 **(E)**, and GPR109A **(F)**. Sample size: *n* = 15 hypertension elderly and 15 hypertension longevity. *Indicates *p* < 0.05.

To further explore the relationship between SCFAs and hypertension, we then directly measured the levels of serum SCFAs and plasma SCFAs receptors. Since no blood samples were collected from the participants, 30 samples (15 in HTE and 15 in HTL) from long-lived populations with hypertension characteristics collected earlier were selected (23). Hypertension long-lived elderly had higher serum acetate levels than hypertension elderly (*p* = 0.011) (Figure 6A). There were no significant differences between the groups’ levels of serum propionate, butyrate (Figures 6B, C), isobutyrate, valerate, and isovalerate. We then quantified the mRNA levels of SCFA-sensing receptors, GPR41, GPR43, and GPR109A, in white blood cells from these samples. There were no changes in the expression of GPR41, GPR43, and GPR019A (Figures 6D–F).

## Discussion

This study is the first cross-sectional study to explore the phenomenon of hypertension in long-lived populations. Microbiome and metabolomic techniques were conducted to characterize the gut microbiota, urine metabolites, and SCFAs of longevity populations with hypertension. The HTE group experienced gut dysbiosis, similar to those reported in hypertensive patients (2, 28), such as higher levels of *Klebsiella* and *Streptococcus*, but not in the HTL group. The HTL group had higher *Bacteroides*, *Faecalibacterium*, and *Alistipes*, which may protect the long-lived elderly against hypertension-induced damage. In addition, the microbial pathways associated with *K. pneumoniae* and *E. coli* may promote hypertension, while *A. muciniphila* may play a role in reversing the development of hypertension in long-lived elderly. Fecal SCFAs levels showed that fecal acetate and propionate in the HTL group were significantly lower, while urine acetate was significantly higher than in the HTE group. Furthermore, HTL exhibited higher serum acetate, but their immune cells expressed no significant changes in SCFAs receptors. In conclusion, our study revealed that hypertension elderly experienced gut dysbiosis, while hypertension long-lived elderly had a unique microbiome and efficient acetate absorption capability in the colon, which can offset the damage of hypertension and maintain healthy homeostasis.

Compared to HTL, the HTE group had significantly higher levels of F/B, and more hypertension-related genera increased in the HTE group, such as *Klebsiella* and *Streptococcus*. F/B has been used to assess the stability of gut microbiota. A higher value indicates a more disordered intestinal flora and a greater the possibility of hypertension. These conclusions have been verified in hypertensive patients (30) and spontaneously hypertensive rats (6). From the characteristics of gut microbiota, compared with the control population, hypertensive patients always have a higher abundance of *Klebsiella*, *Clostridium*, *Streptococcus*, *Porphyromonas*, and *Actinomyces*, and a lower abundance of *Bacteroides*, *Faecalibacterium*, *Roseburia* than the control population (2). In this study, the disturbance of gut microbiota in the HTE group was similar to the previous report, but the HTL group was quite different. The HTL group had higher *Bacteroides*, *Faecalibacterium*, and *Alistipes* than the HTE group. *Bacteroides* and *Alistipes* are important acetate and propionate producing bacteria (31), and *Faecalibacterium* are important butyrate-producing bacteria (32), which are biomarkers to distinguish hypertensive patients in the average population (11). These genera are essential for maintaining health, suggesting that the HTL group has a health-relevant gut microbiome profile.

The search for biomarkers with significant differences between groups (Figure 3) has implications for exploring the phenomenon of hypertension in long-lived people and can explore the relationship between gut microbiota and hypertension at the species level. *Bacteroides*, *F*. *prausnitzii*, *Parabacteroides*, and *Alistipes* were enriched in HTL. *F.prausnitzii* can produce butyrate, which affects blood pressure through vasodilation or plasminogen activator inhibitor-1 (33). *Alistipes* are a producer of acetate and propionate and play a protective role in cardiovascular diseases (34). *Alistipes finegoldii* belonging to the genus *Alistipes* is a species that protects the integrity of the intestinal barrier against colitis (35), which contributes to the absorption of energy substances in the colon. Compared to the HEL group, the HTE had higher levels of *Klebsiella pneumoniae*, and *Streptococcus salivarius*, a further extension of the genus-level species (*Klebsiella* and *Streptococcus*) reported in previous studies (4). The severity of hypertension was associated with an increased abundance of these two species (36). The enrichment of *K. pneumoniae* directly contributes to blood pressure elevation and hypertension pathogenesis, and it causes intestinal damage, fecal metabolic changes, and renal shifts may be integrated mediators (23).

The correlation analysis showed that *Klebsiella pneumoniae* and *Escherichia coli* might be associated with the microbial pathways of hypertension, while *Akkermansia muciniphila* was the opposite. As we know, *E. coli* is an opportunistic pathogen, the most abundant intestinal flora in the longevity population in this study, which was reported to be associated with the development of hypertension during follow-up (37). Moreover, both *E. coli* and *K. pneumoniae* have been reported to be significantly enriched in carotid atherosclerosis and hypertension patients, which generated lipopolysaccharide (LPS) (38) and Trimethylamine (TMA) (39). LPS can be transferred to the bloodstream when the intestinal barrier function is impaired, causing asymptomatic hypoendotoxemia (40). TMA is converted by liver enzymes to TMAO, which causes cardiovascular damage (41) and exacerbates angiotensin II-induced hypertension (42). Based on these findings, we inferred that *E. coli* and *K*. *pneumoniae* have similar microbial pathways and may be involved in the development of hypertension. On the contrary, the abundance of *A. muciniphila* is negatively associated with obesity, type 2 diabetes, and hypertension (43, 44). Therefore, as a new generation of probiotics, the microbial pathway of *A.muciniphila* is significantly different from that of *K*. *pneumoniae* and *E. coli.* It may play a role in reversing the development of hypertension, and its specific mechanism needs further exploration.

Besides gut microbiota, the changes in SCFAs were also correlated to blood pressure (45). The concentrations of fecal acetate and propionate in the HTL group were significantly lower, while urine acetate was significantly higher than HTE. Furthermore, HTL exhibited higher serum acetate, but their immune cells expressed no significant changes in SCFAs receptors. A common explanation is that the lower fecal SCFAs in the long-lived elderly may be due to the reduction of SCFA-producing bacteria and the decreased metabolic activity of the gut microbiota (46). However, the relative abundance of SCFA-producing bacteria such as *Bacteroides*, *Faecalibacterium*, and *Alistipes* was higher in HTL than in HTE, and there was no significant difference in dietary fiber intake between the two groups, so it would not be lower from the perspective of SCFAs production. Everything gets interesting when explained in terms of SCFA uptake levels. SCFAs are efficiently absorbed in the colon, and less than 5% are excreted in the feces. Fecal SCFAs reflect SCFAs that cannot be absorbed in the host colon (3) and are surrogate indicators of colonic SCFAs absorption. Fecal SCFAs concentrations are more representative of SCFAs uptake than their production (8). Fecal SCFAs are significantly increased, and serum SCFAs are significantly decreased in hypertensive patients, indicating that the adequate absorption level of SCFAs in hypertensive patients is lower (11). Higher levels of fecal SCFAs are associated with gut dysbiosis, obesity, and hypertension, presumably due to the lower absorption rate of SCFAs in the gut (47). In contrast, participants with lower blood pressure had lower levels of fecal SCFAs and higher levels of SCFA-producing bacteria, which may promote intestinal absorption of SCFAs (5). Above all, these SCFA-producing bacteria have two functions: they produce SCFAs and promote the absorption of SCFAs in the human intestine, with their activity contributing to the maintenance of low levels of fecal SCFAs and higher levels of blood SCFAs and even urine of the host.

More importantly, we noticed that the acetate level in the HTL group was lower in the fecal and higher in the blood and urine compared to the HTE group. It is important to note that most SCFAs are consumed in the gastrointestinal tract and liver. The remaining SCFAs (mainly acetate) can be transported to other organs *via* the bloodstream to regulate the host’s metabolism, physiology and energy balance (48). Acetate and propionate both enter the portal venous circulation and peripheral blood, but they play different roles. The propionate is mainly used for glycoisogenesis in the liver, which is then metabolized to CO_2_ through the tricarboxylic acid cycle and other metabolic pathways. In contrast, acetate enters the systemic circulation and reaches peripheral tissues, which activates signaling *via* binding to 3 G-protein–coupled receptors, with GPR43 being the most predominant of these receptors (9). These receptors are highly expressed in immune cells, including T, B, and innate lymphoid cells, and activate anti-inflammatory downstream pathways to decrease blood pressure (49). The high absorption of acetate in the colon and more urine excretion of the HTL group supports the hypothesis that the HTL group have unique gut microbiota and efficient acetate absorption capacity, which can offset hypertension damage and thus maintain healthy homeostasis.

Although this research used a relatively small sample size and did not include a healthy control group, our study took advantage of the only longevity and hypertension cohort published to date with well-characterized BP monitoring in both men and women, all untreated for BP-lowering medication. This study is also the only cohort with detailed information regarding diet, urine metabolites, plasma, fecal SCFAs, and their receptors, which allowed us to explore the interplay between the gut microbiome, diet, urine metabolites, SCFAs, and their receptors regard to longevity. Future studies should expand the sample size and use ambulatory BP monitoring to minimize intra-individual variation in samples. Meanwhile, due to the limitation of 16S rRNA sequencing, future studies will use metagenome to uncover the species that play a crucial role in the association between longevity and hypertension.

## Conclusion

In conclusion, we found that people in longevity areas of Guangxi, China, generally with hypertension. Hypertension elderly had much higher levels of hypertension-related bacteria *Klebsiella* and *Streptococcus*, while hypertension long-lived elderly had higher levels of short-chain fatty acid-producing bacteria (*Bacteroides*, *Faecalibacterium*, *Alistipes*). Moreover, *K. pneumoniae* and *E. coli* may play a role in promoting hypertension, while *A. muciniphila* may play a role in reversing the development of hypertension. SCFAs further suggest that acetate can be effectively absorbed in the colon of long-lived elderly with hypertension to offset the negative effects of hypertension. An important mechanism may be driven by SCFA-producing bacteria and their main metabolite, acetate, associated with hypertension longevity. The enrichment of SCFA-producing bacteria, such as *Bacteroides spp*, *Faecalibacterium spp*, and *Alistipes spp* may promote efficient acetate absorption in the colon and represent a new target for BP therapy in the future.

## Data availability statement

The original contributions presented in the study are publicly available. This data can be found here: https://www.ncbi.nlm.nih.gov/bioproject/PRJNA888351.

## Ethics statement

The studies involving human participants were reviewed and approved by the Medical Ethics Committee, Guangxi University. The patients/participants provided their written informed consent to participate in this study.

## Author contributions

QL, ZQ, and QZ conceived and designed the experiments. QZ, NM, YL, HZ, RL, KH, HL, XY, and JM contributed sample collection, investigation, and data analysis. QZ wrote the original draft. QL, DH, ZZ, YL, and ZQ contributed to review and edit. QL, ZQ, and YL contributed to final approval of the publication. QL, ZQ, YL, and QZ are accountable for all the aspects of the work. All authors have read and agreed to the published version of the manuscript.
