# Supplementary material for: Protection effect of gut microbiota composition and acetate absorption against hypertension-induced damages on the longevity population in Guangxi, China
Source: Front Nutr. 2023 Jan 16;9:1070223. doi: 10.3389/fnut.2022.1070223 (PMC9884688; doi:10.3389/fnut.2022.1070223)
Supplement: Supplementary file 1 [file Data_Sheet_1.docx]

**Frontiers in Nutrition**

**Protection effect of gut microbiota composition and acetate absorption against hypertension-induced damages on the longevity population in Guangxi, China**

Qinren Zhang ^1^, Ning Meng ^1^, Yu Liu ^2^, Haiyan Zhao ^2^, Zhengtao Zhao ^1^, Dan Hao ^3^, Ruiding Li ^1^, Kunchen Han ^1^, He Li ^1^, Jinke Ma ^1^, Xiaohan Yu ^1^, Zhongquan Qi ^2,^*, Quanyang Li ^1,^*

1. College of Light Industry and Food Engineering, Guangxi University, Nanning 530004, China
2. Medical College, Guangxi University, Nanning 530004, China
3. Department of Pharmacology and Nutritional Science, University of Kentucky, Lexington, KY, United States

* Correspondence:

E-mail:[liquanyang@gxu.edu.cn](mailto:liquanyang@gxu.edu.cn) (Quanyang Li), [yxyyz@gxu.edu.cn](mailto:yxyyz@gxu.edu.cn) (Zhongquan Qi)

**Figures**


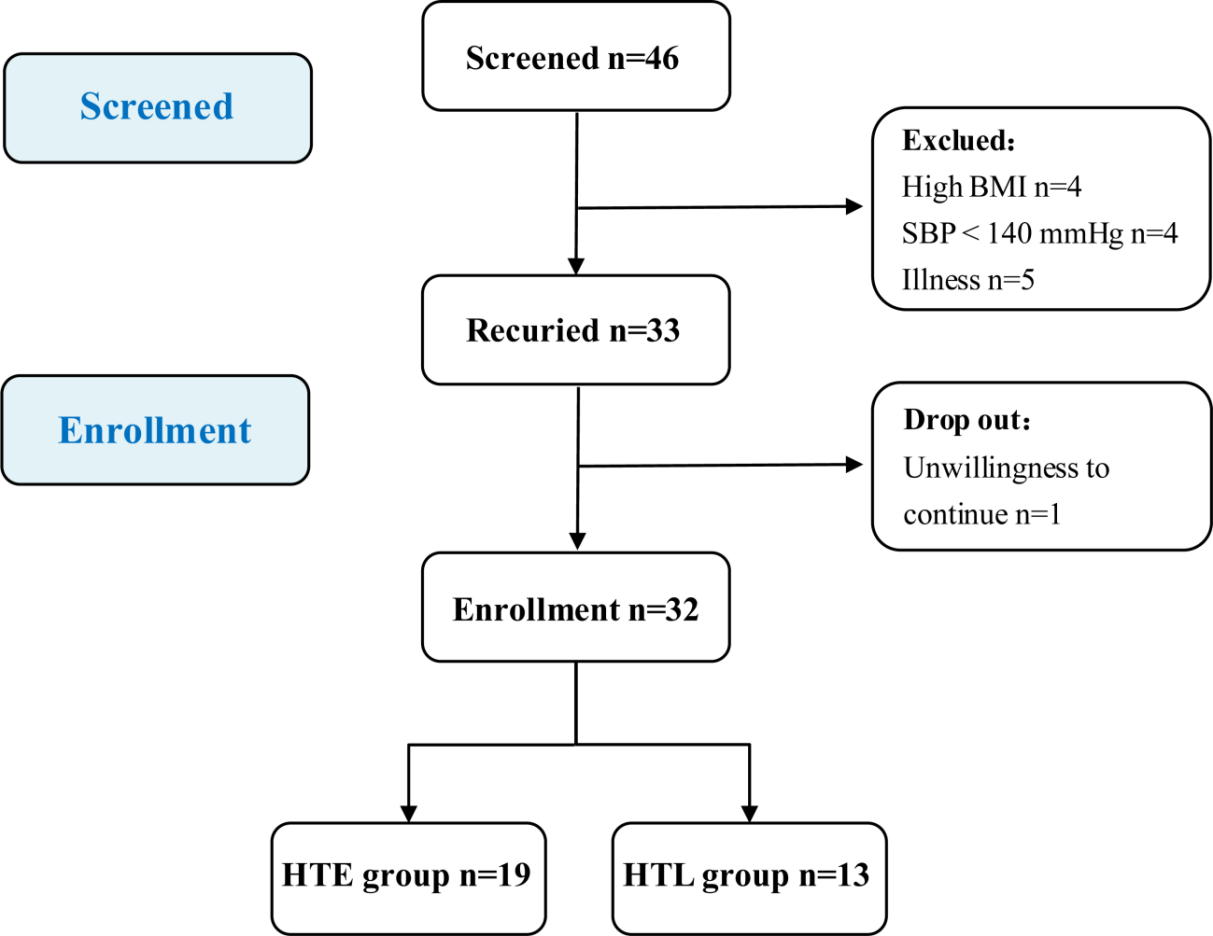


**Figure S1** Flow diagram of participants during the study


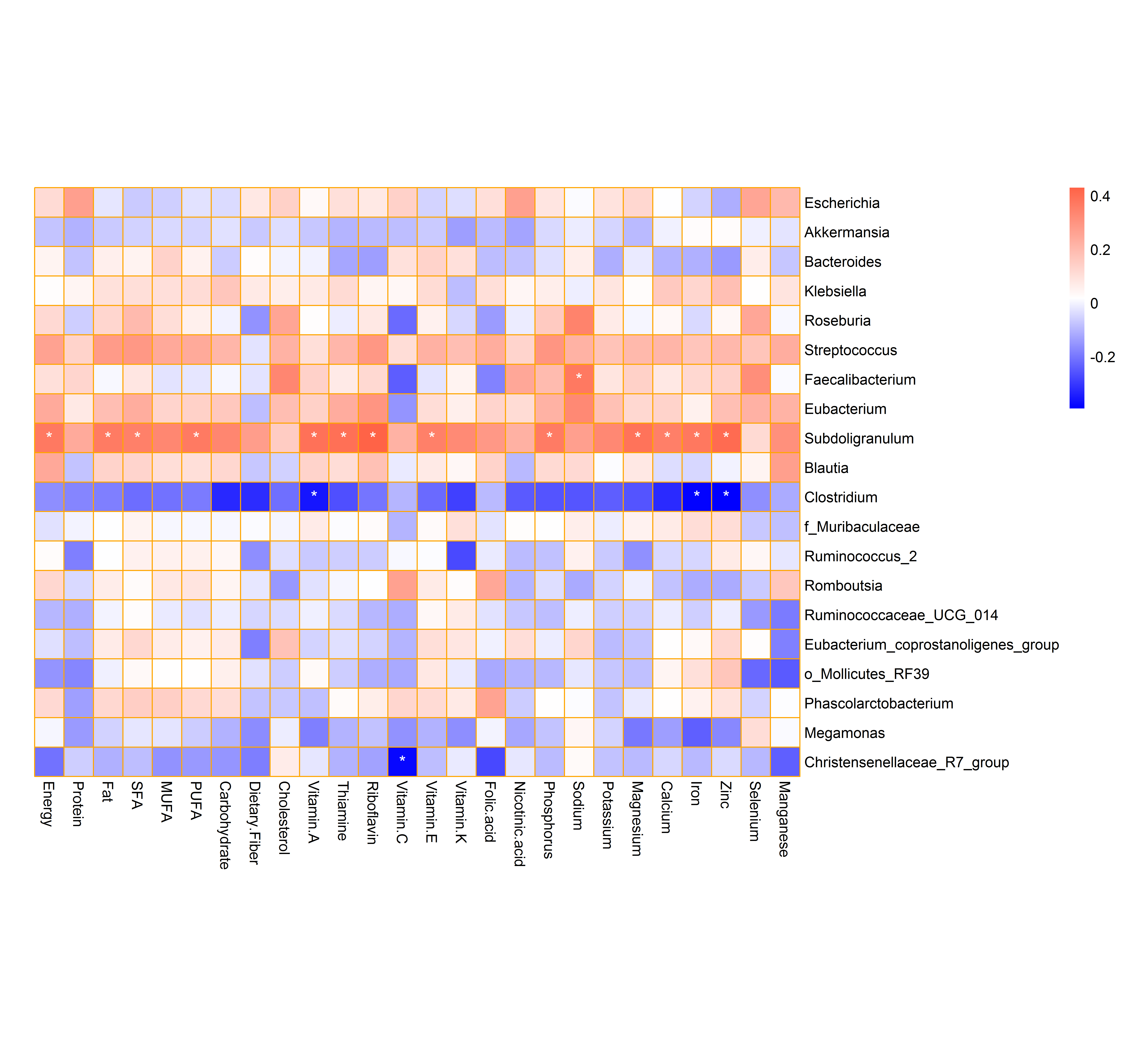


**Figure S2** Heat map of Spearman correlation between nutrient intake and top 20 abundance genus

**
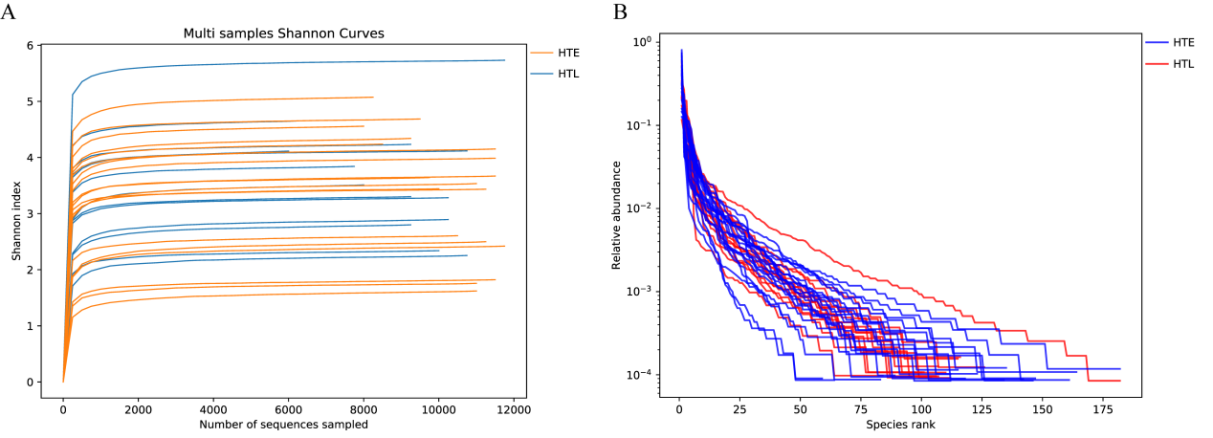
**

**Figure S3** Basic characteristics of all sequenced samples. (A) Shannon curve; (B) rank abund curve


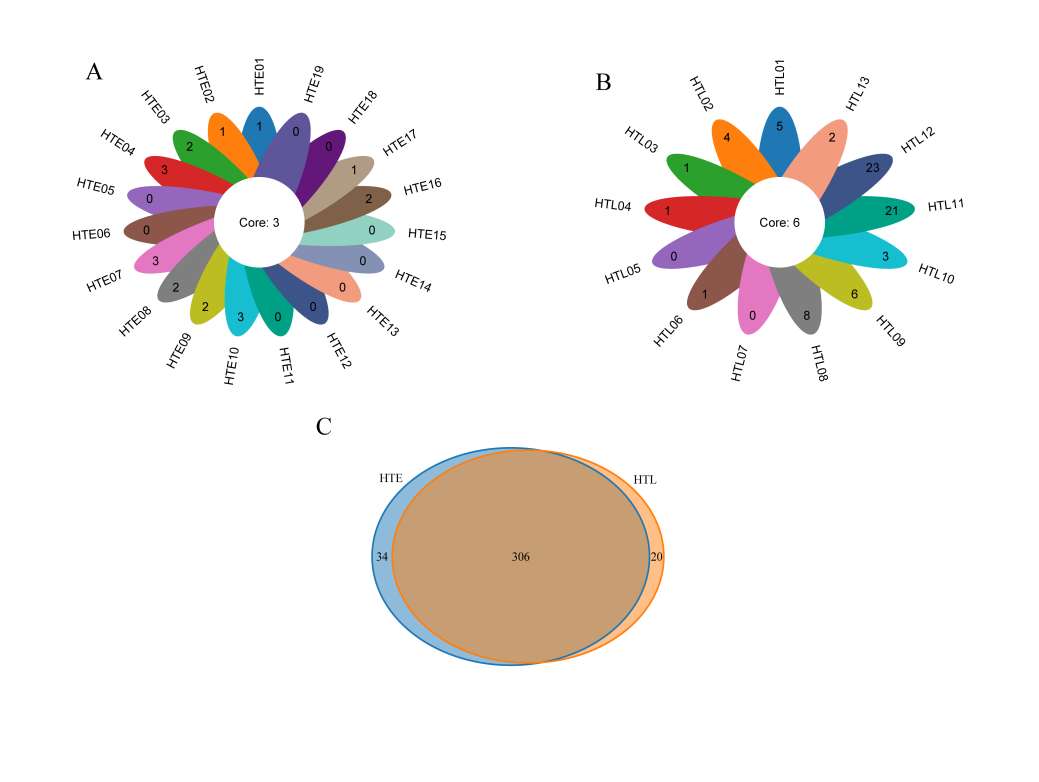


**Figure S4** Venn diagrams of the distribution of OTU characteristics. The OTU characteristic of HTE(A) and HTL(B); The OTU characteristic within and between groups.

**
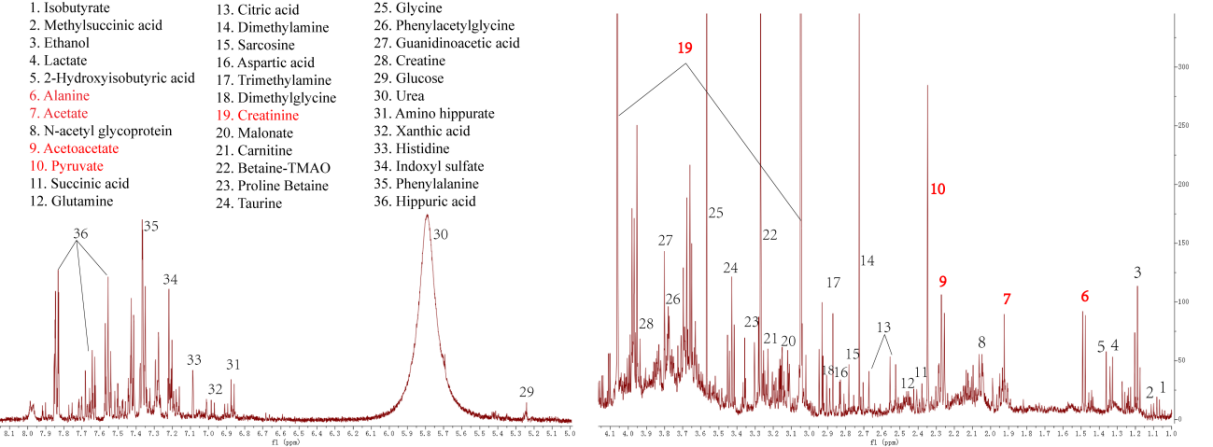
**

**Figure S5** Representative ^1^H-NMR spectrum of urine sample. The figure shows 36 identified metabolites, and the red marks 5 significantly different metabolites with significant differences (*p*<0.05) through Mann-Whitney test. For high spectral clarity, only the peaks of metabolic groups in the same metabolite are labeled, and the water peak (4.2-5.0ppm) has been excluded.

**Tables**

Table S1 The health conditions of the subjects

| Subjects | Sex | Age | Health conditions | | | | | |
| --- | --- | --- | --- | --- | --- | --- | --- | --- |
|  |  |  | Hypertension | Blurred vision | Lumbar disc herniation | Osteoproliferation | Drug use | Eating ability |
| HTE01 | Female | 60 | yes | no | no | no | 0 | yes |
| HTE02 | Male | 60 | yes | no | no | no | 0 | yes |
| HTE04 | Male | 62 | yes | no | no | no | 0 | yes |
| HTE03 | Male | 62 | yes | no | no | no | 0 | yes |
| HTE05 | Female | 63 | yes | no | no | no | 0 | yes |
| HTE06 | Male | 64 | yes | no | no | no | 0 | yes |
| HTE08 | Male | 66 | yes | no | no | no | 0 | yes |
| HTE07 | Female | 66 | yes | no | no | no | 0 | yes |
| HTE09 | Male | 69 | yes | no | no | yes | 1 | yes |
| HTE10 | Male | 70 | yes | no | no | no | 0 | yes |
| HTE12 | Male | 70 | yes | no | no | no | 0 | yes |
| HTE13 | Male | 70 | yes | no | no | no | 0 | yes |
| HTE11 | Female | 70 | yes | no | no | no | 0 | yes |
| HTE14 | Female | 75 | yes | no | no | no | 0 | yes |
| HTE15 | Female | 76 | yes | no | no | no | 0 | yes |
| HTE16 | Female | 79 | yes | no | no | no | 0 | yes |
| HTE17 | Male | 84 | yes | no | no | no | 0 | yes |
| HTE18 | Female | 85 | yes | no | no | no | 0 | yes |
| HTE19 | Female | 88 | yes | no | no | no | 0 | yes |
| HTL01 | Female | 92 | yes | no | no | no | 0 | yes |
| HTL02 | Male | 93 | yes | no | no | no | 0 | yes |
| HTL03 | Male | 94 | yes | no | no | no | 0 | yes |
| HTL04 | Female | 94 | yes | no | no | no | 0 | yes |
| HTL05 | Female | 98 | yes | no | no | no | 0 | yes |
| HTL06 | Male | 100 | yes | no | no | no | 0 | yes |
| HTL07 | Male | 101 | yes | no | no | no | 0 | yes |
| HTL08 | Female | 102 | yes | no | no | no | 0 | yes |
| HTL09 | Female | 102 | yes | yes | yes | no | 0 | no or little^1^ |
| HTL10 | Female | 105 | yes | no | no | no | 0 | yes |
| HTL11 | Female | 105 | yes | no | no | no | 0 | yes |
| HTL12 | Female | 108 | yes | yes | no | no | 0 | yes |
| HTL13 | Female | 109 | yes | no | no | no | 0 | yes |

^1^ "no or little help" identifies subjects who could need help in cutting or spreading food.

Table S2 Strengthening The Organization and Reporting of Microbiome Studies (STORMS) reporting checklist.

| **Number** | **Item** | **Yes/No/NA** | **Comments or location in manuscript** |
| --- | --- | --- | --- |
| **Abstract** |  |  |  |
| 1 | Structured or Unstructured Abstract | Yes | Abstract |
| 1.1 | Study Design | Yes | Abstract |
| 1.2 | Sequencing methods | Yes | Abstract |
| 1.3 | Specimens | Yes | Abstract |
| **Introduction** |  |  |  |
| 2 | Background and Rationale | Yes | Page 2 and 3 |
| 2.1 | Hypotheses | Yes | Page 3 |
| **Methods** |  |  |  |
| 3 | Study Design | Yes | Page 3 and 4 (Participant recruitment and study groups) |
| 3.1 | Participants | Yes | Page 3 and 4 (Participant recruitment and study groups) |
| 3.2 | Geographic location | Yes | Page 3 and 4 (Participant recruitment and study groups) |
| 3.3 | Relevant Dates | Yes | Page 3 and 4 (Participant recruitment and study groups) |
| 3.4 | Eligibility criteria | Yes | Page 3 and 4 (Participant recruitment and study groups) |
| 3.5 | Antibiotics Usage | Yes | Page 3 and 4 (Participant recruitment and study groups) |
| 3.6 | Analytic sample size | Yes | Figure S1 |
| 3.7 | Longitudinal Studies | NA |  |
| 3.8 | Matching | NA |  |
| 3.9 | Ethics | Yes | Page 3 and 4 (Participant recruitment and study groups) |
| 4 | Laboratory methods | Yes | Page 4 (16S rRNA third-generation full-length sequencing of gut microbiota) |
| 4.1 | Specimen collection | Yes | Page 4 (16S rRNA third-generation full-length sequencing of gut microbiota) |
| 4.2 | Shipping | Yes | Page 4 (16S rRNA third-generation full-length sequencing of gut microbiota) |
| 4.3 | Storage | Yes | Page 4 (16S rRNA third-generation full-length sequencing of gut microbiota) |
| 4.4 | DNA extraction | Yes | Page 4 (16S rRNA third-generation full-length sequencing of gut microbiota) |
| 4.5 | Human DNA sequence depletion or  microbial DNA enrichment | NA |  |
| 4.6 | Primer selection | Yes | Page 4 (16S rRNA third-generation full-length sequencing of gut microbiota) |
| 4.7 | Positive Controls | Yes | Page 4 (16S rRNA third-generation full-length sequencing of gut microbiota) |
| 4.8 | Negative Controls | NA |  |
| 4.9 | Contaminant mitigation and identification | Yes | Page 4 (16S rRNA third-generation full-length sequencing of gut microbiota) |
| 4.10 | Replication | Yes | Page 4 (16S rRNA third-generation full-length sequencing of gut microbiota) |
| 4.11 | Sequencing strategy | Yes | Page 4 (16S rRNA third-generation full-length sequencing of gut microbiota) |
| 4.12 | Sequencing methods | Yes | Page 4 (16S rRNA third-generation full-length sequencing of gut microbiota) |
| 4.13 | Batch effects | NA |  |
| 4.14 | Metatranscriptomics | NA |  |
| 4.15 | Metaproteomics | NA |  |
| 4.16 | Metabolomics | Yes | Pages 5 and 6 (Fecal short-chain fatty acid measurement and Analysis of urine metabolite based on ^1^H-Nuclear Magnetic Resonance) |
| 5 | Data sources/ measurement | Yes | Pages 3 and 4 (Participant recruitment and study groups, Dietary Assessment) |
| 6 | Research design for causal inference | Yes | Pages 6 (Statistics and analysis) |
| 6.1 | Selection bias | NA |  |
| 7 | Bioinformatic and Statistical  Methods | Yes | Page 4 (16S rRNA third-generation full-length sequencing of gut microbiota) |
| 7.1 | Quality Control | Yes | Page 4 (16S rRNA third-generation full-length sequencing of gut microbiota) |
| 7.2 | Sequence analysis | Yes | Page 4 (16S rRNA third-generation full-length sequencing of gut microbiota) |
| 7.3 | Statistical methods | Yes | Pages 4 (16S rRNA third-generation full-length sequencing of gut microbiota, Statistics and analysis) |
| 7.4 | Longitudinal analysis | NA |  |
| 7.5 | Subgroup analysis | NA |  |
| 7.6 | Missing data | NA |  |
| 7.7 | Sensitivity analysis | NA |  |
| 7.8 | Findings | Yes | Pages 4 and 6 (16S rRNA third-generation full-length sequencing of gut microbiota, Statistics and analysis) |
| 7.9 | Software | Yes | Pages 4 and 6 (16S rRNA third-generation full-length sequencing of gut microbiota, Statistics and analysis) |
| 8 | Reproducible research | Yes | Pages 4 and 6 (16S rRNA third-generation full-length sequencing of gut microbiota, Statistics and analysis) |
| 8.1 | Raw data access | Yes | Pages 4 and 6 (16S rRNA third-generation full-length sequencing of gut microbiota) |
| 8.2 | Processed data access | No | We have provided access to all raw data. |
| 8.3 | Participant data access | No | Individual participant data cannot be provided. |
| 8.4 | Source code access | Yes | https://www.ncbi.nlm.nih.gov/bioproject/PRJNA888351 |
| 8.5 | Full results | No | Values have been inputed into Tables in the supplementary files as they are easier to read. |
| **Results** |  |  |  |
| 9 | Descriptive data | Yes | Page 4 and 5 (Table 1 and Table 2) |
| 10 | Microbiome data | Yes | Pages 7 (Analysis of gut microbiota characteristics between group) |
| 10.1 | Taxonomy | Yes | Pages 7 (Analysis of gut microbiota characteristics between group) |
| 10.2 | Differential abundance | Yes | Pages 7 (Analysis of gut microbiota characteristics between group) |
| 10.3 | Other data types | Yes | Pages 7, 8 and 9 (Analysis of gut microbiota characteristics between group, Hypertension-related microbial microbial pathways) |
| 10.4 | Other statistical analysis | Yes | Pages 8 and 9 (Fecal short-chain fatty acid levels, Statistical analysis of urine metabolites, SCFAs and Receptors) |
| **Discussion** |  |  |  |
| 11 | Key results | Yes | Pages 9 (first paragraph) |
| 12 | Interpretation | Yes | Pages 10-13 |
| 13 | Limitations | Yes | Pages 13 |
| 13.1 | Bias | No |  |
| 13.2 | Generalizability | Yes | Pages 13 |
| 14 | Ongoing/future work | Yes | Pages 13 |
| **Other information** |  |  |  |
| 15 | Funding | Yes | Pages 13 (Funding) |
| 15.1 | Acknowledgements | Yes | Pages 13 (Acknowledgments) |
| 15.2 | Conflicts of Interest | Yes | Pages 14 (Conflict of Interest) |
| 16 | Supplements | Yes | Attached, journal will link them |
| 17 | Supplementary data | Yes | Attached, journal will link them |

Table S3 Basic characteristics of the 30 subjects involved in the study

|  | **HTE** | **HTL** | ***p-*value** |
| --- | --- | --- | --- |
| Male/female | 9/6 | 2/13 | - |
| Age | 66.87±9.68 | 99.53±5.28 | <0.001 |
| BMI | 22.18±2.57 | 20.03±1.87 | 0.019 |
| SBP | 148.00±10.83 | 156.67±15.76 | 0.067 |
| DBP | 83.37±10.96 | 83.37±11.33 | 0.838 |

All values are presented as mean±SD.

Table S4 The three main receptors used in the qPCR.

| **Genes** | **Primer** |
| --- | --- |
| FFAR3(GPR41) | Forward: 5’-TTCACCACCATCTATCTCACCG-3’  Reverse: 5’-GGAACTCCAGGTAGCAGGTC-3’ |
| FFAR2(GPR43) | Forward: 5’-TGCTACGAGAACTTCACCGAT-3’  Reverse: 5’-GGAGAGCATGATCCACACAAAAC-3’ |
| HCAR2(GPR109A) | Forward: 5’-ATGTTGGCTATGAACCGCCAG-3’  Reverse: 5’-GCTGCTGTCCGATTGGAGA-3’ |
| GDAPH(housekeeping gene) | Forward: 5’-GGAGTCCACTGGCGTCTTC-3’  Reverse: 5’-GGTTCACACCCATGACGAAC-3’ |

Table S5 Alpha diversity comparison between HTE and HTL.

| α diversity | **HTE** | **HTL** | ***p-*value** |
| --- | --- | --- | --- |
| Feature | 119.37±7.81 | 113.08±6.36 | 0.362 |
| ACE | 138.48±8.67 | 129.21±5.77 | 0.383 |
| Chao1 | 139.50±9.01 | 130.29±5.69 | 0.305 |
| Simpson | 0.73±0.04 | 0.77±0.41 | 0.570 |
| Shannon | 3.46±0.24 | 3.63±0.27 | 0.970 |
| PD_whole_tree | 10.01±0.55 | 9.64±0.46 | 0.448 |

All values are presented as mean±SD.

Table S6 Bacterial genera altered in hypertension elderly with their classification

| HTE | Phylum | Genus | *p*-value | References |
| --- | --- | --- | --- | --- |
| Up regulate | *Proteobacteria* | *Klebsiella* | 0.005 | (1) |
|  | *Firmicutes* | *Ruminococcus* | 0.018 | (2) |
|  | *Firmicutes* | *Streptococcus* | 0.059 | (3) |
| Down regulate | *Bacteroidetes* | *Bacteroides* | 0.033 | (4) |
|  | *Firmicutes* | *Faecalibacterium* | 0.030 | (3, 5) |
|  | *Bacteroidetes* | *Parabacteroides* | 0.037 | (5) |
|  | *Bacteroidetes* | *Alistipes* | <0.001 | New |
|  | *Bacteroidetes* | *Erysipelotrichaceae_UCG-003* | 0.041 | New |

Table S7 Metabolic pathways with significant differences between the HTE and the HTL.

| **KEGG pathway** | **Class** | **Mean relative abundance(%)** | | ***p*-value** |
| --- | --- | --- | --- | --- |
|  |  | **HTE** | **HTL** |  |
| ABC transporters | Environmental Information Processing；Membrane transport | 3.38 | 2.98 | 0.022 |
| Alanine, aspartate and glutamate metabolism | Metabolism;  Amino acid metabolism | 0.84 | 0.82 | 0.004 |
| Arginine biosynthesis | Metabolism; Amino acid metabolism | 0.46 | 0.50 | 0.005 |
| Benzoate degradation | Metabolism;  Xenobiotics biodegradation and metabolism | 0.19 | 0.12 | 0.045 |
| Degradation of aromatic compounds | Metabolism;  Global and overview maps | 0.23 | 0.15 | 0.037 |
| GABAergic synapse | Organismal Systems; Nervous system | 0.10 | 0.13 | 0.001 |
| Glycosaminoglycan degradation | Metabolism;  Glycan biosynthesis and metabolism | 0.09 | 0.12 | 0.014 |
| Inositol phosphate metabolism | Metabolism; Carbohydrate metabolism | 0.13 | 0.12 | 0.027 |
| Lysosome | Cellular Processes; Transport and catabolism | 0.11 | 0.19 | 0.016 |
| Other glycan degradation | Metabolism;  Glycan biosynthesis and metabolism | 0.18 | 0.29 | 0.003 |
| Phosphotransferase system (PTS) | Environmental Information Processing；  Membrane transport | 0.59 | 0.39 | 0.027 |
| Propanoate metabolism | Metabolism; Carbohydrate metabolism | 0.62 | 0.56 | 0.024 |
| Quorum sensing | Cellular Processes;  Cellular community - prokaryotes | 1.33 | 1.25 | 0.041 |
| Selenocompound metabolism | Metabolism; Metabolism of other amino acids | 0.33 | 0.31 | 0.013 |
| Sphingolipid metabolism | Metabolism; Lipid metabolism | 0.16 | 0.22 | 0.024 |
| Tyrosine metabolism | Metabolism; Amino acid metabolism | 0.21 | 0.17 | 0.037 |

Table S8 Urine metabolites significantly related to blood pressure

| **Urine Metabolites** | **SBP** | | **DBP** | |
| --- | --- | --- | --- | --- |
|  | **Correlation** | ***p*-value** | **Correlation** | ***p*-value** |
| Glucose | - | - | 0.382* | 0.037 |
| Taurine | - | - | 0.397* | 0.030 |
| Carnitine | - | - | 0.444 | 0.014 |
| Creatinine | -0.391* | 0.033 | - | - |
| Sarcosine | - | - | -0.525* | 0.003 |
| Citric acid | - | - | -0.466* | 0.009 |
| N-acetyl glycoprotein | -0.366* | 0.046 | -0.516* | 0.003 |
| Methylsuccinic acid | -0.421* | 0.021 | - | - |

The results were adjusted by age and BMI * *p*-value < 0.05; ** *p*-value < 0.01; - means no significant difference.

**References:**

1. Li J, Zhao F, Wang Y, Chen J, Tao J, Tian G, et al. Gut microbiota dysbiosis contributes to the development of hypertension. MICROBIOME. 2017;5(1).

2. Sun S, Lulla A, Sioda M, Winglee K, Wu MC, Jacobs DR, et al. Gut Microbiota Composition and Blood Pressure. HYPERTENSION. 2019;73(5):998-1006.

3. Yan Q, Gu Y, Li X, Yang W, Jia L, Chen C, et al. Alterations of the Gut Microbiome in Hypertension. FRONT CELL INFECT MI. 2017 2017-08-24;7.

4. Kim S, Rigatto K, Gazzana MB, Knorst MM, Richards EM, Pepine CJ, et al. Altered Gut Microbiome Profile in Patients With Pulmonary Arterial Hypertension. HYPERTENSION. 2020;75(4):1063-71.

5. Calderón-Pérez L, Gosalbes MJ, Yuste S, Valls RM, Pedret A, Llauradó E, et al. Gut metagenomic and short chain fatty acids signature in hypertension: a cross-sectional study. SCI REP-UK. 2020;10(1).
